# Supplementary material for: Anti-Inflammatory, Anti-Oxidative and Anti-Apoptotic Effects of Thymol and 24-Epibrassinolide in Zebrafish Larvae
Source: Antioxidants (Basel). 2023 Jun 18;12(6):1297. doi: 10.3390/antiox12061297 (PMC10294864; doi:10.3390/antiox12061297)
Supplement: Supplementary file 1 [file antioxidants-12-01297-s001.zip › antioxidants-2437521-supplementary.pdf]

Supplementary material

Figure S1 - Graph with the number of neutrophils migrated to the site of injury after 4 hpi, in the different tested concentrations of THY (A) and 24-EPI (B), value was normalised according to the control group (Mean of control: THY: 29.2 ± 5.5; 24-EPI: 32.9 ± 7.2). Data are expressed as mean ± SD and statistical analysis was performed using a one-way ANOVA followed by Tukey's multiple comparison test. Different letters represent statistical differences among treatment groups ($p < 0.05$).

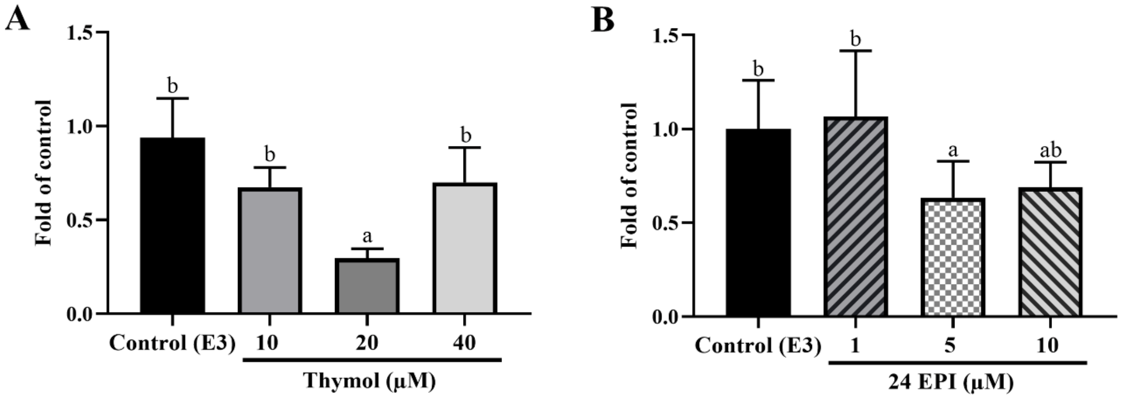

Table S1 - Normalised biochemical markers (fold of control) evaluated in zebrafish WT larvae with 72 hpf, exposed for 4 hours to different treatments.

Biochemical Markers	Treatment groups								Statistical test	p
	Control (E3)	$CuSO_4$ 10 µM	DIC 1.5 µM	$CuSO_4$+DIC	24-EPI 5 µM	$CuSO_4$+24EPI	THY 20 µM	$CuSO_4$+THY		
**GST**	1.0 ± 0.1	0.8 ± 0.1	1.0 ± 0.2	0.9 ± 0.1	0.9 ± 0.1	0.8 ± 0.09	0.8 ± 0.1	0.8 ± 0.2	F (7,38) = 1.122	0.370
**GSH**	1.0 ± 0.1	0.9 ± 0.1	0.8 ± 0.1	0.9 ± 0.1	0.8 ± 0.1	0.8 ± 0.1	0.7 ± 0.1	0.7 ± 0.06	F (7,38) = 1.610	0.162
**GSSG**	1.0 ± 0.1	0.9 ± 0.1	0.6 ± 0.1	1.0 ± 0.2	0.7 ± 0.2	0.8 ± 0.04	0.9 ± 0.2	0.8 ± 0.2	F (7,38) = 2.022	0.077
**LPO**	1.0 ± 0.2	0.7 ± 0.1	1.1 ± 0.1	0.8 ± 0.4	1.0 ± 0.3	1.0 ± 0.06	1.0 ± 0.2	0.8 ± 0.4	F (7,38) = 0.957	0.476

Data from at least five independent replicates of 50 animals each, is expressed as mean ± SD for parametric data distribution (Mean of control: GST= 17.7 ± 2 (µmol DNPH/min mg protein); GSH = 136.88 ± 26.3 (µmol GSH/mg protein); GSSG = 858.5 ± 375.8 (µmol GSSG/mg protein); LPO = 2 184 ± 1 562 (mmol MDA/mg protein)). Values were normalised to the control group. Statistical analysis was performed using one-way ANOVA followed by Tukey's multiple-comparison test or Kruskal-Wallis followed by Dunn’s test. Different letters indicate significant statistical differences between groups ($p < 0.05$).
